# The Association between Dietary Patterns and Depressive Symptoms in Chinese Adults

**DOI:** 10.1155/2020/8380151

**Published:** 2020-08-29

**Authors:** Mingyuan Zhang, Zhijun Li, Shuman Yang, Yaoyao Sun, Mengdi Jin, Xin Chen, Qiong Yu

**Affiliations:** ^1^Cancer System Biology Center, China-Japan Union Hospital, Jilin University, Changchun 130033, China; ^2^Department of Epidemiology and Biostatistics, School of Public Health, Jilin University, Changchun 130021, China

## Abstract

**Background:**

Previous studies of the relationship between diet and depression have focused on single nutrients or food. Recent research suggested that dietary patterns may offer more information than an individual nutrient in assessing disease risk. We designed this study to assess the association between dietary patterns and depressive symptoms in the adult population of China.

**Methods:**

We identified 372 Chinese residents for this research. Factor analysis was used to extract dietary patterns from 30 predefined food groups. Dietary intake was assessed using an effective self-administered food frequency questionnaire, and depressive symptoms were assessed using the 9-item Patient Health Questionnaire (PHQ-9) score. Subjects were considered to have depressive symptoms when they had a PHQ-9 score of >4.

**Results:**

We identified four eating patterns: “vegetables-fruits,” “traditional Chinese,” “pastry-fruits,” and “animal food” dietary patterns. After adjusting for potential confounders, participants in the highest tertile animal food pattern (considered to be an unhealthy pattern) were more prone to depressive symptoms compared with participants in the lowest tertile (OR = 2.08, 95% CI: 1.02-4.24).

**Conclusions:**

The animal food pattern was associated with an increased risk of depressive symptoms.

## 1. Introduction

Depression has become an important public health problem worldwide [1]. Depression is the fourth cause of disability in the world in 2000; it will be the second leading cause of disease burden by 2020 [2]. It is quite important to determine modifiable risk factors for depression.

Recently, diet has been considered to be an adjustable factor for depression [3]. The relationship between depressive symptoms and specific nutrients and foods, such as vitamin C [4], vitamin D [5, 6], folate [7], and fish [8, 9], is inconsistent. That is to say, for the study of dietary factors, researchers initially focused on the effects of an individual nutrient. However, a lot of nutrients are highly correlated with each other, and some nutrients may also affect intestinal absorption of other nutrients [10]. Therefore, studying the overall dietary pattern analysis to examine the complex relationship between diet and disease risk has begun to become popular. There are two main methods to define dietary patterns. The first method is to use dietary records or food frequency questionnaire (FFQ) data to derive dietary patterns from statistical models (such as factor analysis). Second, a hypothesis-oriented approach that uses predefined criteria to construct dietary pattern scores can be used [11]. A meta-analysis by Lassale et al. [12] included a total of 20 longitudinal studies and 21 cross-sectional studies that utilized an array of dietary measures to study the relationship between dietary patterns and depression outcomes.

Until now, the data on the relationship between dietary patterns and depressive symptoms in China are insufficient. Only three studies have examined the association of dietary patterns with depressive symptoms of adults in Chinese; their conclusions are not consistent [3, 13, 14]. Therefore, using a cross-sectional study across 19 provinces in China, we examined the relationship between dietary patterns and depressive symptoms.

## 2. Materials and Methods

### 2.1. Participants

The dietary survey was collected in January 2018, which covered 19 provinces in China. Since we have plans to continue the follow-up, we chose convenient sampling. The inclusion criteria for the survey participants were (1) aged 30 years or older at the interview, (2) able to give consent to participate in the study, and (3) capable of understanding and answering the questions. The participants were identified and interviewed face-to-face by medical students in Jilin University (enrolment year: 2017; specialty: preventive medicine) using a structured questionnaire. The participants for this survey included the relatives, friends, and neighbors of the students. A total of 400 questionnaires were distributed, and 372 valid questionnaires were returned. After excluding subjects with missing dietary information and subjects lacking any variable information in the main analysis, 266 subjects (32–95 years old, 133 males and 133 females) remained to analyze the dietary patterns and the relationship between dietary patterns and depressive symptoms. All subjects gave their informed consent for inclusion before they participated in the study. The study was conducted in accordance with the Declaration of Helsinki, and the protocol was approved by the Ethics Committee of the School of Public Health, Jilin University (approval number: 2016-03-013).

### 2.2. Depressive Symptoms

Depressive symptoms were assessed using the 9-item Patient Health Questionnaire (PHQ-9) score [15], which was incorporated into the lifestyle questionnaire. PHQ-9 is one of the most common self-assessment screening tools for depression and has shown acceptable screening efficacy in a lot of populations, including Chinese patients in primary care settings [16–18]. The scale is composed of 9 questions, each question is scored on a scale of 1-3 according to the frequency of symptoms, and the total PHQ-9 score ranges from 0 to 27 [15]. Subjects were considered to have depressive symptoms when they had a PHQ-9 score of >4.

### 2.3. Dietary Assessment

Each participant was asked to fill out a questionnaire that included the food item information, the size of each portion, and the average frequency of consumption per day, week, month, and year for the past year. The researchers explained the size of each portion to the participants using a picture catalogue of individual food portions. Foods were divided into 30 predefined food groups based on similar nutrients and biological origins, which were used to derive dietary patterns by factor analysis.

### 2.4. Covariates

The research mainly included the dietary information of the respondents and general demographic characteristics such as age, body mass index (BMI), sex, and educational background. The educational background was classified into two categories: ≤9 years or >9 years. Marriage status was classified as married or single. The smoking status (“current smoker,” “former smoker,” and “never smoker”), drinking status (“yes” or “no”), and nap situation (“yes” or “no”) were obtained through questionnaires.

### 2.5. Statistical Analysis

We entered extracted food groups into the factor analysis and used the principal component method to determine the number of factors or dietary patterns. Each factor was rotated using orthogonal transformation (varimax rotation) to keep the factors uncorrelated and have better interpretability. Based on the eigenvalues, the scree test, and the interpretability of the factors, we determined the number of factors to retain. There were twelve factors that satisfied the criteria for eigenvalues > 1. The scree plots dropped significantly (from 1.62 to 1.40) after the fourth factor and remained similar after the fifth factor (1.40 for the fifth and 1.35 for the sixth factor); we decided to retain four factors. Individuals with missing data from more than ten dietary items were excluded from the factor analysis; for those who missed ten or fewer items, it was assumed that the missing items were not consumed and were given the value of 0 [19]. We named dietary patterns based on three factors of food items showing high loading (absolute value). Each dietary pattern and each individual's factor scores were calculated by summing intakes of food items weighted by their factor loadings.

The difference in proportions and means of covariates according to depressive symptoms was assessed by using the *χ*^2^ test and independent *t*-test, respectively. The trend test was assessed by entering tertiles of each dietary pattern score in all models, distributing ordinal numbers 1-3 to tertile categories of each dietary pattern. We performed logistic regression analysis to estimate the odds ratio and 95% confidence intervals. We adjusted (1) age and (2) age, BMI, sex, educational background, marital status, afternoon nap, drinking status, and smoking status. All statistical analyses were performed using the statistical package SPSS version 24.0 (SPSS Inc., Illinois, USA).

## 3. Results

As shown in Table 1, four dietary patterns were identified in this study according to the results of factor analysis. The first factor, identified as the vegetables-fruits pattern, was characterized by a great consumption of vegetables, fruits, flour, aquatic products, egg, and nuts. For the second factor, we named it as the traditional Chinese pattern, because it represented high intakes of cereals, vegetables, fruits, aquatic products, beans, and nuts. The third factor represented high intakes of pastry, buckwheat, millet, various kinds of fruits, beef, aquatic products, egg products, and nuts, and we named the pattern as the pastry-fruits pattern. The fourth factor was typified by a high consumption of rice, sugarcane, root vegetables, cabbage vegetables, pork, beef, chicken, other meat, beans, aquatic products, egg, and tea; thus, it was named as the animal food pattern. The first to fourth dietary patterns accounted for 18.3%, 6.7%, 5.6%, and 5.4% of the variance in food intakes, respectively, and fully explained 36.0% of the variability.

The characteristics of the study subjects according to their depressive symptoms are shown in Table 2. There were 85 (32%) participants who were identified as having depressive symptoms (PHQ-9 scale scores of >4) among 266 participants included in the cross-section analysis. Compared with subjects who did not have depressive symptom, those who had depressive symptoms were younger (*P* = 0.011). In addition, smoking status was different between subjects with and without depressive symptoms (*P* = 0.009). After post hoc multiple comparison, it was found that current smokers tended to have depressive symptoms.

Table 3 shows the characteristics according to tertile categories of dietary pattern scores. Participants with a higher score of the vegetables-fruits pattern appeared to be nonalcohol users (*P* = 0.020). The subjects with a higher score of the traditional Chinese pattern were more likely to have an afternoon nap (*P* = 0.024). A higher pastry-fruits pattern score was associated with the lower education level (*P* < 0.001) and BMI (*P* = 0.015). The animal food pattern was significantly associated with sex (*P* = 0.001), drinking status (*P* = 0.020), and smoking status (*P* = 0.003). Those who had a higher score of the animal food pattern were more likely to be male, to drink alcohol, and to be current smokers.

The ORs for depressive symptoms associated with the tertile categories of each dietary pattern score are shown in Table 4. In an age-adjusted model (model 1), the high animal food pattern score was significantly associated with increased prevalence of depressive symptoms. After adjusting for other covariates, this association was still statistically significant (OR = 2.08, 95% CI: 1.02-4.24, and *P* = 0.043). In the aged-adjusted (model 1) and fully adjusted models (model 2), other dietary patterns were not associated with depressive symptoms.

## 4. Discussion

Using a sample of the Chinese adult population, we identified four major dietary patterns via factor analysis: traditional Chinese, vegetables-fruits, pastry-fruits, and animal food patterns. The results showed that a high intake of the animal diet pattern was associated with an increased risk of depressive symptoms. There was a significant and positive association between the depressive symptom score and the animal diet pattern.

In our study, there were no significant associations of depressive symptoms with the vegetables-fruits pattern and the traditional Chinese pattern. Although the two dietary patterns are not the same, they both are mainly composed of a large number of different types of vegetables and fruits. These results are inconsistent with previous studies, in which eating patterns with large amounts of vegetables, fruits, and fish reduced the risk of depressive symptoms [20, 21]. Two Japanese studies have shown that a healthy Japanese dietary pattern characterized by large intakes of plant foods, including fruits, vegetables, mushrooms, and soy products, plays a critical role in preventing depressive symptoms [22, 23]. On the one hand, some studies have shown that higher levels of antioxidants are associated with lower risk of depression; high levels of antioxidants in fruits and vegetables may have protective effects [24, 25]. On the other hand, research has shown that lower levels of folic acid increase the risk of depression [26]. The potential protective effect of a healthy dietary pattern on depression was attributed to folic acid which is identified in many cruciferous vegetables, green leafy vegetables, other green vegetables, and dried legumes [24]. Therefore, the biological effects of these two patterns on depressive symptoms seemed to be reasonable, though we did not observe a link between them. The lack of association between these two patterns and depressive symptoms in this study could be due to differences in the cooking method. Chinese people prefer to eat cooked vegetables, which may lead to the loss of some antioxidant contents contained in fruits and vegetables [27].

In terms of health outcomes, in this study, we found a positive correlation between animal food patterns and depressive symptoms through error bar graphs and multivariate regression analysis of adjusted covariates. In our study, people with unhealthy diets were more prone to depressive symptoms, which was consistent with previous studies [28–30]. In fact, there are several explanations for this result. First, previous studies have shown that large amounts of red meat were associated with a high risk of cardiovascular disease and inflammation, and cardiovascular disease and inflammation are involved in the pathogenesis of depression [31, 32]. In addition, large consumption of red meats and processed meats that contain lots of saturated fatty acids was associated with higher levels of low-grade inflammation (C-reactive protein) and subsequent brain atrophy, which are positively associated with depression [33].

We did not find a significant association between the pastry-fruits pattern and the depressive symptoms; this dietary pattern was rarely reported in previous studies. This pattern was made up of the healthy and unhealthy food groups. Pastries contain a lot of sugar, and epidemiological studies have shown that large intakes of sugar altered endorphin levels and oxidative stress in the body, which was significantly associated with the increased risk of depression [34]. At the same time, the pastry-fruits pattern also includes a large number of fruits and aquatic products. According to previous reports, fruits and aquatic products were protective factors of depressive symptoms [20, 22]. The study of dietary patterns assesses the effect of the overall diet on depressive symptoms, while the pastry-fruits pattern of dietary patterns consists of a complex combination of food and nutrition; this may explain this null association to some extent. In addition, the possibility of reverse causation may not be ruled out. People with depression may change their eating behavior and food choices, either adopting an unhealthy diet (i.e., high-calorie foods) or reducing food intake [28, 35].

Our results were not completely consistent with the results of the previous three studies in China [3, 13, 14]. We only found the association between the animal-food pattern and the depressive symptoms. Considering that the dietary pattern is culturally specific and the food groups selected by each study are not exactly the same, future research is warranted to confirm this association. Overall, in our study, the association between depression scores and diverse dietary patterns was investigated. We concluded that the animal food pattern was associated with depressive symptoms.

There are some potential limitations worth considering. First, this study is a cross-sectional study that fails to allow for the causal relationship between the dietary patterns and the risk of depressive symptoms. In addition, the study used a semiquantitative food questionnaire that covered only specific foods, not as accurate as the dietary assessment of the diary questionnaire. At the same time, although the PHQ-9 has been widely recognized, it is a self-reported form after all, and there may still be several errors in the classification of the results. Finally, although we have managed to control some suspicious confounding factors, we still cannot rule out the potential effect of other unmeasured factors.

Based on the results of this study and meta-analysis by Lassale et al. [12], most current studies focus on depressive symptoms and lack of evidence of clinical depression.

## 5. Conclusions

Our research suggests that the animal food pattern is associated with an increased risk of depressive symptoms. Given that the dietary habits of the Chinese population are not completely consistent with those of other countries, further cohort studies are needed to confirm our findings in the future.

## Figures and Tables

**Table 1:** Factor analysis varimax-rotated factor loading scores^1^ of 30 food groups.

	Vegetables-fruits pattern	Traditional Chinese pattern	Pastry-fruits pattern	Animal food pattern
Rice and rice products	0.02	-0.01	0.02	*0.46*
Wheat flour and products	*0.39*	*0.20*	*-0.23*	-0.02
Other cereals and products^2^	-0.03	*0.80*	*0.21*	-0.02
Potato	0.01	*0.82*	0.03	-0.10
Mixed beans	-0.10	0.16	0.19	0.01
Pastry	0.04	0.14	*0.77*	0.17
Citrus fruits	*0.38*	*0.20*	*0.36*	-0.05
Kernel fruits	*0.34*	*0.58*	*0.35*	-0.11
Stone fruits	*0.37*	-0.08	*0.46*	-0.13
Small fruits, berries	0.04	0.05	*0.65*	-0.04
Tropical fruits (peel edible)	*0.43*	*-0.25*	0.14	0.00
Other fruits^3^	*0.21*	*0.28*	-0.03	*0.37*
Solanaceous fruits	*0.44*	0.15	*0.30*	0.15
Melon vegetables	*0.67*	0.14	0.06	0.14
Onion and garlic	*0.77*	0.03	0.15	0.11
Stem vegetables	*0.54*	0.13	0.09	0.10
Root vegetables	*0.54*	*0.26*	-0.13	*0.25*
Cabbage vegetables	*0.43*	*0.44*	-0.02	*0.20*
Leafy vegetables	0.12	*0.27*	0.10	0.10
Pickled vegetables	*0.21*	*0.51*	-0.01	0.06
Pork	0.16	-0.01	0.10	*0.72*
Beef	-0.09	0.13	*0.41*	*0.51*
Chicken	0.05	0.06	0.16	*0.62*
Other meat	0.11	-0.10	-0.06	*0.30*
Beans and soy products	0.15	*0.35*	0.04	*0.31*
Milk and dairy products	0.12	0.03	0.17	-0.01
Aquatic products	*0.36*	*0.23*	*0.35*	*0.24*
Egg and egg products	*0.28*	0.16	*0.35*	*0.33*
Nuts	*0.37*	*0.34*	*0.20*	0.09
Tea	-0.01	0.04	-0.11	*0.37*

^1^Factor loading greater than ±0.2 is shown in italic. ^2^Included buckwheat and millet. ^3^Included sugarcane.

**Table 2:** Characteristics of subjects with and without depressive symptoms (*n* = 266)^1^.

Characteristics	Subjects without depressive symptoms (*n* = 181)		Subjects with depressive symptoms (*n* = 85)		*P* value^2^
	Mean (*n*)	SD (%)	Mean (*n*)	SD (%)	
Age (years)	54.5	(10.0)	51.3	(9.0)	0.011
BMI (kg/m^2^)	23.5	(2.8)	23.8	(3.1)	0.476
Sex					
Male	89	(49.2)	44	(51.8)	0.693
Female	92	(50.8)	41	(48.2)	
Educational background					
≤9 years	97	(53.6)	36	(42.4)	0.087
>9 years	84	(46.4)	49	(57.6)	
Marital status					
Married	165	(91.2)	72	(84.7)	0.115
Single	16	(8.8)	13	(15.3)	
Afternoon nap					
Yes	94	(51.9)	40	(47.1)	0.458
No	87	(48.1)	45	(52.9)	
Drinking status					
Yes	68	(37.6)	34	(40.0)	0.704
No	113	(62.4)	51	(60.0)	
Smoking status					
Current smoker	23	(12.7)	23	(27.1)	0.009
Former smoker	21	(11.6)	12	(14.1)	
Never smoker	137	(75.7)	50	(58.8)	

^1^Participants with a PHQ-9 score of >4 were judged as depressed. ^2^*P* values of categorical variables (chi-squared test) and of continuous variables (independent *t*-test).

**Table 3:** Characteristics according to tertile categories of dietary pattern scores.

	Vegetables-fruits pattern			Trend *P*^1^	Traditional Chinese pattern			Trend *P*^1^	Pastry-fruits pattern			Trend *P*^1^	Animal food pattern			Trend *P*^1^
	Low	Middle	High		Low	Middle	High		Low	Middle	High		Low	Middle	High	
Age (years)	54.4 ± 10.6	52.4 ± 9.5	53.7 ± 9.3	0.645	53.5 ± 10.1	53.3 ± 9.3	53.6 ± 10.0	0.890	54.3 ± 10.4	53.5 ± 8.6	52.6 ± 10.4	0.249	54.6 ± 10.1	53.0 ± 8.9	53.0 ± 10.4	0.269
BMI (kg/m^2^)	23.7 ± 3.0	23.2 ± 2.7	23.8 ± 3.1	0.865	23.3 ± 3.2	23.6 ± 3.1	23.9 ± 2.5	0.191	24.1 ± 3.2	23.5 ± 3.0	23.1 ± 2.6	0.015	23.1 ± 2.8	23.8 ± 2.9	23.8 ± 3.1	0.082
Sex																
Male	61.4	38.9	50.0	0.132	53.4	42.2	54.5	0.880	48.9	46.7	54.5	0.452	40.9	43.3	65.9	0.001
Female	38.6	61.1	50.0		46.6	57.8	45.5		51.1	53.3	45.5		59.1	56.7	34.1	
Educational background																
≤9 years	48.9	51.1	50.0	0.880	53.4	55.6	40.9	0.098	69.3	48.9	31.8	<0.001	53.4	50.0	46.6	0.367
>9 years	51.1	48.9	50.0		46.6	44.4	59.1		30.7	51.1	68.2		46.6	50.0	53.4	
Marital status																
Married	86.4	90.0	90.0	0.334	89.8	86.7	90.9	0.809	90.9	91.1	85.2	0.227	88.6	92.2	86.4	0.629
Single	13.6	10.0	9.1		10.2	13.3	9.1		9.1	8.9	14.8		11.4	7.8	13.6	
Afternoon nap																
Yes	54.5	45.6	51.1	0.652	44.3	45.6	61.4	0.024	45.5	50.0	55.7	0.176	48.9	47.8	54.5	0.452
No	45.5	54.4	48.9		55.7	54.4	38.6		54.5	50.0	44.3		51.1	52.2	45.5	
Drinking status																
Yes	45.5	41.1	28.4	0.020	36.4	41.1	37.5	0.877	31.8	41.1	42.0	0.164	31.8	34.4	48.9	0.020
No	54.5	58.9	71.6		63.6	58.9	62.5		68.2	58.9	58.0		68.2	65.6	51.1	
Smoking status																
Current smoker	22.7	15.6	13.6	0.242	22.7	13.3	15.9	0.329	18.2	16.7	17.0	0.205	12.5	13.3	26.1	0.003
Former smoker	10.2	12.2	14.8		10.2	14.4	12.5		18.2	13.3	5.7		11.4	7.8	18.2	
Never smoker	67.0	72.2	71.6		67.0	72.2	71.6		63.6	70.0	77.3		76.1	78.9	55.7	

^1^On the basis of linear regression analysis for continuous variables and the Mantel–Haenszel *χ*^2^ test for categorical variables, the ordinal numbers 1-3 were assigned to the tertile category of each dietary pattern.

**Table 4:** Odds ratios and 95% CIs for depressive symptoms according to tertiles of dietary pattern scores.

	Prevalence rate (%)	Model 1^1^		Model 2^2^	
		OR (95% CI)	*P* value	OR (95% CI)	*P* value^3^
Traditional Chinese pattern					
Low	9.0	Reference		Reference	
Middle	12.0	1.39 (0.73-2.65)	0.318	1.50 (0.75-3.00)	0.249
High	11.0	1.30 (0.67-2.49)	0.436	1.42 (0.71-2.84)	0.324
Vegetables-fruits pattern					
Low	9.8	Reference		Reference	
Middle	13.2	1.53 (0.81-2.88)	0.187	1.70 (0.86-3.33)	0.126
High	9.0	0.90 (0.46-1.75)	0.755	0.96 (0.48-1.94)	0.912
Pastry-fruits pattern					
Low	9.8	Reference		Reference	
Middle	12.4	1.36 (0.72-2.57)	0.344	1.34 (0.68-2.64)	0.394
High	9.8	0.93 (0.48-1.81)	0.840	0.85 (0.40-1.78)	0.657
Animal food pattern					
Low	7.1	Reference		Reference	
Middle	11.7	1.83 (0.93-3.60)	0.078	1.94 (0.96-3.92)	0.063
High	13.2	2.30 (1.18-4.51)	0.015	2.08 (1.02-4.24)	0.043

^1^Adjusted for age. ^2^Adjusted for age, BMI, sex, educational background, marital status, afternoon nap, drinking status, and smoking status. ^3^Based on the multiple logistic regression analysis, assigning ordinal numbers 1-3 to tertile categories of each dietary pattern.

## Data Availability

The datasets generated or analyzed during the current study are available from the corresponding author (Qiong Yu, email: yuqiong@jlu.edu.cn) upon reasonable request.
